# Clustering Field-Based Maize Phenotyping of Plant-Height Growth and Canopy Spectral Dynamics Using a UAV Remote-Sensing Approach

**DOI:** 10.3389/fpls.2018.01638

**Published:** 2018-11-13

**Authors:** Liang Han, Guijun Yang, Hao Yang, Bo Xu, Zhenhai Li, Xiaodong Yang

**Affiliations:** ^1^School of Geoscience and Surveying Engineering, China University of Mining and Technology-Beijing, Beijing, China; ^2^College of Architecture and Geomatics Engineering, Shanxi Datong University, Datong, China; ^3^Key Laboratory of Quantitative Remote Sensing in Agriculture of Ministry of Agriculture, Beijing Research Center for Information Technology in Agriculture, Beijing, China; ^4^National Engineering Research Center for Information Technology in Agriculture, Beijing, China; ^5^Beijing Engineering Research Center for Agriculture Internet of Things, Beijing, China

**Keywords:** unmanned aerial vehicles, high-throughput phenotyping platform, time series clustering, breeding, development, maize, typical curve

## Abstract

Phenotyping under field environmental conditions is often considered as a bottleneck in crop breeding. Unmanned aerial vehicle high throughput phenotypic platform (UAV-HTPP) mounted with multi-sensors offers an efficiency, non-invasive, flexible and low-cost solution in large-scale breeding programs compared to ground investigation, especially where measurements are time-sensitive. This study was conducted at the research station of the Xiao Tangshan National Precision Agriculture Research Center of China. Using the UAV-HTPP, RGB and multispectral images were acquired during four critical growth stages of maize. We present a method of extracting plant height (PH) at the plot scale using UAV-HTPP based on the spatial structure of the maize canopy. The core steps of this method are segmentation and spatial Kriging interpolation based on multiple neighboring maximum pixels from multiple plants in a plot. Then, the relationships between the PH extracted from imagery collected using UAV-HTPP and the ground truth were examined. We developed a semi-automated pipeline for extracting, analyzing and evaluating multiple phenotypic traits: canopy cover (CC), normalized vegetation index (NDVI), PH, average growth rate of plant height (AGRPH), and contribution rate of plant height (CRPH). For these traits, we identify genotypic differences and analyze and evaluate dynamics and development trends during different maize growth stages. Furthermore, we introduce a time series data clustering analysis method into breeding programs as a tool to obtain a novel representative trait: typical curve. We classified and named nine types of typical curves of these traits based on curve morphological features. We found that typical curves can detect differences in the genetic background of traits. For the best results, the recognition rate of an NDVI typical curve is 59%, far less than the 82.3% of the CRPH typical curve. Our study provides evidence that the PH trait is among the most heritable and the NDVI trait is among the most easily affected by the external environment in maize.

## Introduction

Numerous studies have shown that global food production must be doubled by 2050 to meet the rising demand. However, the output of the world’s major food crops is not growing at this rate (Ray et al., 2013). Increasing crop yield by breeding is an effective means to solve the global food security problem. To meet the future need and accelerate progress in breeding for novel traits, plant breeders wish to phenotype large numbers of lines rapidly and accurately to identify the best progeny (Araus and Cairns, 2014). However, phenotyping under field environmental conditions is often considered as a bottleneck in crop breeding (Cobb et al., 2013; Yang et al., 2017).

To break this bottleneck, over the past few years, several field-based, high throughput phenotyping platforms (HTPPs) have been applied to successfully measure phenotypic traits for different crop breeding, such as soybean (Bai et al., 2016), wheat (Crain et al., 2016), cereals (Busemeyer et al., 2013), and cotton (Andrade-Sanchez et al., 2013). These ground-based HTPPs are assembled by modified vehicles, proximity sensors and other devices. They have the advantages of high resolution, flexible design and large payload. However, these ground-based HTPPs do have several limitations in terms of scale, efficiency, and redeployment in different fields, which increase the cost of promotion (Haghighattalab et al., 2016). Taking maize as an example, due to the plant height (PH) restrictions, its use is not very feasible, except for during the early growth stages (Montes et al., 2011). In recent years, unmanned aerial vehicle high-throughput phenotypic platforms (UAV-HTPPs) have gradually become a powerful tool for acquiring field crop phenotypic information, with advantages of maneuverability, suitability for a complex farmland environment, high operational efficiency, non-invasiveness and low cost (Yang et al., 2017). UAV-HTPPs have been applied to different crops, such as Haghighattalab et al. (2016) and Holman et al. (2016) for wheat; Chapman et al. (2014) and Potgieter et al. (2017) for sorghum; and Shi et al. (2016) for maize, sorghum, and winter wheat.

Different phenotypic traits can be acquired using UAV-HTPPs mounted with different types of cameras. Some of the cameras used include RGB, multispectral, hyperspectral, and thermal cameras and airborne LiDAR (light detection and ranging). Consumer level digital RGB cameras allow estimation of green biomass, senescence, and yield (Sakamoto et al., 2012; Casadesus and Villegas, 2014). Multispectral or hyperspectral images can be formulated using different spectral indices for pigment degradation, senescence evaluation, photosynthetic efficiency, nutrient status or water content (Gutierrez et al., 2010; Fritsche-Neto et al., 2015). Thermal images allow measurement of plant water status for phenotyping in the context of water-stress (Jones et al., 2009). LiDAR sensors can directly measure the three-dimensional distribution of plant canopies and estimate PH and canopy structure (Bouvier et al., 2015; Roussel et al., 2017).

High throughput phenotyping techniques in crop breeding are generally used to screen for architectural and physiological traits and early detection of desirable genotypes (Shakoor et al., 2017). Normalized difference vegetation index (NDVI) can be considered as a leaf greenness indicator for trait detection and phenotyping (Walter et al., 2015). NDVI is closely correlated with stay-green and senescence (Liebisch et al., 2015; Duan et al., 2017a). As senescence is a dynamic process, genotypes with different senescence patterns may exhibit a similar final NDVI (Christopher et al., 2014). By acquiring multi-temporal NDVI maps, analysis of senescence dynamics allows for improvement of genotypic stay-green variation discernment. NDVI has also been applied to study crop phenology detection (Lunetta et al., 2009; Verbesselt et al., 2010; De Moura et al., 2017) and environmental stress feedback (Stagakis et al., 2012; Behmann et al., 2014). At the plot scale, an NDVI map derived from UAV-HTPPs includes both plant and soil information; thus, removing soil background from the images can improve the results (Liebisch et al., 2015; Duan et al., 2017a). Canopy cover (CC) traits can be used to assess temporal and genotypic differences and to correlate important plant traits used for crop breeding such as early vigor and senescence (Walter et al., 2015). At the plot scale, CC simply refers to the ratio of plant area to the total area. CC can be calculated from an RGB image or an NDVI map by classification or band operation (Ritchie et al., 2010; Geipel et al., 2014; Duan et al., 2017b). PH is one of the most heritable and easily measured traits in maize (Peiffer et al., 2014). Breeders often select dwarfed cultivars to reduce lodging, select taller plants to produce bioenergy, or select suitable parental lines for hybrid breeding (Barmeier et al., 2016). PH can be obtained from multi-temporal crop surface models (CSMs) derived from three-dimensional (3D) point clouds generated from UAV-HTPP images using structure from motion (SFM) techniques (Westoby et al., 2012). Several studies have already used CSM to estimate PH for different crops including maize (Geipel et al., 2014), barley (Bendig et al., 2015), wheat (Holman et al., 2016), and sorghum (Watanabe et al., 2017).

Turner et al. (2012) found that ground control points (GCPs) can provide significant spatial accuracy improvements. Using the method of CSM with GCPs, Roth and Streit (2017) reported a strong and significant overall correlation of PH to ground truth values measured using a meter stick during the different growth stages. At the plot scale, CSM mixed with soil and plant pixels can lead to underestimation of PH, which has not been sufficiently addressed, especially for small-plot and low-density planting maize breeding programs.

Although the use of an UAV-HTPP has been successfully demonstrated in many experiments, many studies have been limited to a single date. In addition, the dynamic development and the relationship between plant growth and environmental variables will form an important focus of the next generation phenotyping (Walter et al., 2015). Actually, UAV-HTPPs not only can provide single measurements to evaluate traits but they can also combine multi-temporal trait datasets to assess the dynamics of plant growth and development. Liebisch et al. (2015) used a Zeppelin airship as a remote-sensing platform to acquire RGB, NDVI and thermal images throughout the maize growing season and to analyze the seasonal development of CC, NDVI and canopy temperature among 16 maize genotypes. Clear differences between genotypes were detected for these traits, but no detailed discussion was given.

Cluster analysis is an exploratory data analysis tool in agronomy which aims to group and select different agronomic traits in such a way that the degree of relation between the two traits is maximal if they belong to the same group and minimal otherwise. Clustering is commonly considered as an unsupervised procedure. The definition of clusters depends on the user with obvious subjectivity (Aghabozorgi et al., 2015), and thus clustering evaluations can also be rather subjective. Cluster validity indices (CVIs) can provide standardized cluster evaluation metrics, avoiding subjectivity in the selection of a cluster number (Maulik and Bandyopadhyay, 2002).

The core of time-series clustering analysis is the distance measurement between samples of different time series (Montero and Vilar, 2014). The shape-based distance (SBD) measure derived from the K-shape algorithm was used in this study, because a k-Shape creates homogeneous and well-separated clusters, and outperforms partitional, hierarchical, and spectral clustering approaches (Paparrizos and Gravano, 2016). Dynamic cluster analysis through time series data can assess the development rules and changing rules of traits in the time-space dimension, and provide a comprehensive evaluation of traits.

To date, only a few studies have reported attempts to use UAV-HTPP remote sensing for multiple-trait measurement as well as analyze and evaluate dynamics and development trends of phenotypic traits among large-scale genotypes. This work presents a proof of concept exercise of how a UAV-HTPP mounted with multi-sensors could be used for acquiring field maize phenotyping in a large-scale genotype breeding program. We used a time series data analysis method to analyze, identify and evaluate genotypic differences and dynamic changes in traits during different maize growth stages. More specific objectives in this study include: (1) developing a semi-automated pipeline for extracting, analyzing and evaluating multiple phenotypic traits derived from UAV-HTPP; (2) identifying genotypic differences in dynamics and development trends of traits during different maize growth stages by time series clustering; and 3) discussing some potential problems associated with measurement by UAV-HTPP and the effect of genotype-by-environment interaction on the expression of traits.

## Materials and Methods

### Study Area

**FIGURE 1:**
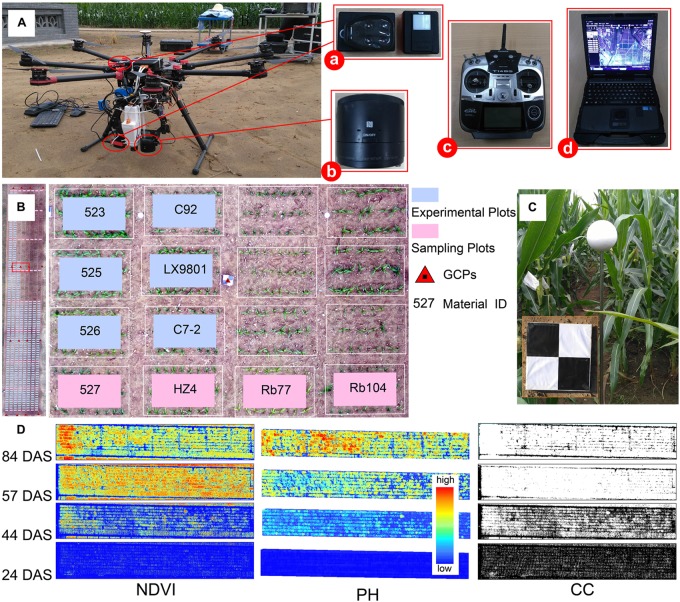
Filed phenotype data collection and multi-temporal image processing results. **(A)** Components of UAV-HTPP: (a) Parrot Sequoia camera (b) Sony Cyber-shot DSC-QX100 digital camera (c) radio control system (d) ground station. **(B)** An overview of the experimental site (487 plots) and part of the plot details. **(C)** GCP markers and plant height reference ball. **(D)** Multi-temporal images of NDVI, PH, and CC. This indicates dynamic change in the trait difference between genotypes. CC, canopy cover; NDVI, normalized difference vegetation index; PH, plant height; DAS, days after sowing.

This study was conducted at the research station of the Xiao Tangshan National Precision Agriculture Research Center of China, which covers an area of approximately 2 km^2^ in Changping District (115°50′17″–116°29′49″E, 40°20′18″–40°23′13″N) of Beijing City. There are relatively flat terrain and homogeneous soil at the experimental field site. To minimize the effect of environmental factors, a single-factor experimental design and the same field management practices were implemented. Meteorological data were acquired from the QT-1060 open-path eddy-covariance systems (Channel Technology Group Limited, China) in the field. Four hundred and eighty-seven maize breeding plots with a size of 2.4 m × 2 m were used to observe phenotypic expression of maize. These plots were planted using a seeding density of 6 plants/m^2^ and a row spacing of 0.6 m (Figure 1B). According to the genetic background difference of maize seeds, these plots were divided into four groups: GRP1 (lower stiffness of stalk), GRP2 (higher stiffness of stalk), GRP3 (longer growing period), GRP4 (mixed group).

### Platform and Image Acquisition

The UAV-HTPP consisted of four parts: UAV, ground station (GS), radio control system (RC), and sensors (Figure 1A). DJI Spreading Wings S1000 (SZ DJI Technology Co., Shenzhen, China) is a low-cost octocopter UAV with a maximum takeoff weight of 10 kg. The eight propeller arms are completely foldable for easier transport and allowing for more power and stability. FUTABA-T14SG RC (Futaba Corp., Chiba, Japan) was used to manually control takeoff and landing of the UAV and to adjust routes in case of emergency. DJI GS was programmed to automatically generate efficient flight paths for UAV and gather images with 80% forward overlap and 75% side overlap at a flight altitude above ground level (AGL) of approximately 40–60 m, on clear days. Each flight speed was set to 6 m per second. ISO and shutter were set to a fixed value (i.e., 160 and 1/2000, respectively).

Equipped with both an optical RGB and a multi-spectral camera, UAV-HTPP collected images simultaneously, including spatial and spectral information during the four critical growth stages. A Sony Cyber-shot DSC-QX100 (Sony Electronics Inc., Tokyo, Japan) and Parrot Sequoia served as optical RGB and multispectral sensors, respectively. In particular, the Parrot Sequoia had another sunshine sensor for radiometric calibration, which recorded the intensity of light emanating from the sun during flight. The Sony RGB camera has a 20.2-megapixel resolution. The Parrot Sequoia camera (MicaSense Inc., Seattle, WA, United States) recorded images in four different spectral bands with the same resolution (1.2 megapixels): green (wavelength 550 nm; bandwidth 40 nm), red (wavelength 660 nm; bandwidth 40 nm), red-edge (wavelength 735 nm; bandwidth 10 nm) and near infrared (wavelength 790 nm; bandwidth 40 nm). To obtain more accurate reflectance values, the radiometric calibration images (RCIs) of the multispectral sensor were captured for a calibrated reflectance panel (MicaSense Inc., Seattle, WA, United States) on the ground before and after each flight.

Sixteen GCPs distributed evenly within the field were used to obtain accurate geographical references on multiple dates, and measured by the position with a differential global positioning system (DGPS, South Surveying and Mapping Instrument Co., Ltd., China) with millimeter accuracy. A reference ball was designed to verify whether the process of PH extraction contains systematic error (Figure 1C). Due to the limitation of the ball’s size and the flight altitude, the reference ball was not clearly identified in the images obtained; thus, these were not effectively used in this study. The details of the UAV data acquisition are listed in Table 1.

**Table 1:** Details of UAV-HTPP image acquisition.

Date	AGL (m)	DAS	Orthomosaic resolution (cm/pix)	DSM resolution (cm/pix)	Point density (points/cm^2^)	GCPs RMSE (cm)
June 8th, 2017	40	24	0.72	1.44	47.9	1.43
June 28th, 2017	60	44	1.33	2.45	14.2	2.17
July 11th, 2017	60	57	1.35	2.47	13.7	2.23
August 7th, 2017	50	84	1.11	2.12	20.2	2.51


*DAS, days after sowing; AGL, flight altitude above ground level; RMSE, root mean square error; DSM, digital surface model; GCPs, ground control points.*

### Developing a Trait-Extraction Pipeline for HTPP

**FIGURE 2:**
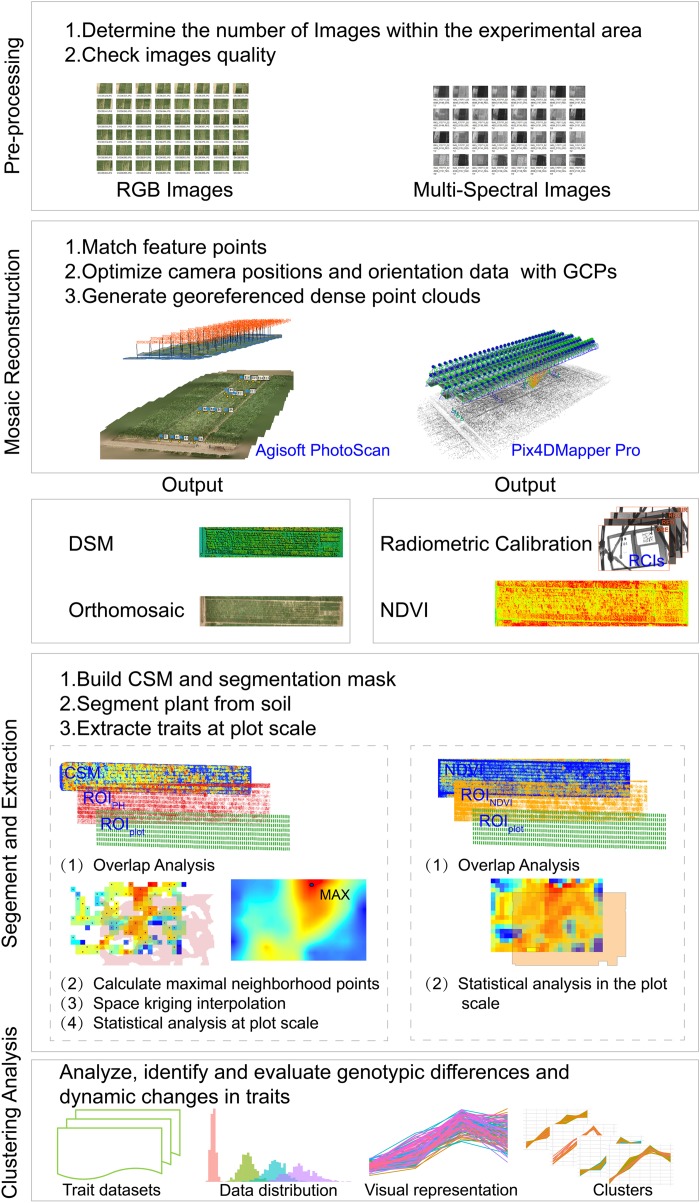
The workflow for extraction, analysis and evaluation the genotypic difference and dynamics and development trend of traits derived from a UAV-HTPP.

To analyze and evaluate the dynamic changes and heterogeneity of CC, PH, and NDVI at the plot scale among different genotypes, we developed a semi-automated image pipeline to acquire, analyze and evaluate the traits extracted from images captured by the UAV-HTPP. This included four main steps: (1) Pre-processing, to check image quality and select usable images. (2) Mosaic reconstruction, to produce digital surface models (DSMs) and NDVI maps. (3) Segmentation and extraction, to divide the image into individual plots, extract the traits from the segmentation result and create trait datasets. (4) Evaluation, to analyze and cluster time-series traits and explain the causes of changes and clustering results. PH and NDVI (and CC) were acquired from optical and multispectral images, respectively. The overall workflow of the developed pipeline is presented in Figure 2, and a detailed description is provided separately.

In the field breeding trials, the field plots were designed to be small and tightly arranged due to the wide variety of maize genotypes and the scarcity of seeds of different genotypes. During the later growth stage, the leaves at the edge of the adjacent plot crossed each other, which could easily cause PH or vegetation index extraction error, so the border of each plot was scaled to the center by 0.25 m. New borders served as masks to clip and save a region of interest (ROIplot) for each plot.

#### Canopy Plant Height

By using structure-from-motion (SFM) software Agisoft PhotoScan 1.3 free trial (Agisoft LLC, St. Petersburg, Russia), DSMs and orthomosaic were produced from optical images shot by UAV with GCPs. This process mainly included feature point matching, dense point cloud generation, DSM and orthomosaic output. Digital elevation model (DEM) (i.e., a non-vegetation ground model) was constructed from the first set of aerial images collected on June 8th by interpolation method. CSMs were calculated by subtracting DSM at different growth stages from DEM. 3D root mean square error (RMSE), point cloud density, orthomosaic resolution and DSM resolution reflected the quality of the mosaiced output during each growth stage (Table 1). CSM is a raster dataset that mixes soils and plants, and two steps were taken to acquire PH at the plot scale.

##### (1) Segment plants from soils

A Normalized Green–Red Difference Index (NGRDI) was used to segment plants from bare soil. NGRDI values for soils are always recorded as negative (Shimada et al., 2012). To create a segmentation mask that contained only plants, image binarization was performed according to positive and negative NGRDI values. NGRDI can be calculated using formula (1) as follows:

$$NGRDI=\frac{\rho_{green}-\rho_{red}}{\rho_{green}+\rho_{red}}$$

ArcGIS spatial Analyst Tools were used to overlay the ROI_plot_ and a segmentation mask to obtain 487 individual ROI_PH_ (ROI of PH), which covered only the plant area of each plot. Extracting the plant pixel from the CSM, we created a new raster dataset CSM_PH_.

##### (2) Calculate the representative value of plant height

Due to the structural characteristics of maize canopy, CSM_PH_ simultaneously covers the imaged pixels from the high and low leaves of multiple plants. Calculating the average value will result in serious PH underestimation. The most ideal method is to calculate the PH by using the imaged pixels in the upper leaves of multiple plants, which requires consideration of the spatial distribution of multiple plants in a plot. We proposed a new solution. First, the CSM_PH_ was resampled to reduce the number of pixels, and split into 3 × 3-pixel windows. Second, the maximum height value in the neighborhood was found to create a set of maximum points. These points had three-dimensional spatial coordinates. Resampling controlled the number of pixels used to calculate the PH at the plot scale. Whether to reduce or increase the number of pixels depends on the spatial resolution of the DSM. Finally, space Kriging interpolation was performed on the points, and the maximum value was taken as a representative value of PH at the plot scale. Using this method, a time series of PH dataset was obtained for later dynamic analysis (Figure 1D).

#### NDVI and Canopy Cover

NDVI Maps were automatically produced by using Pix4DMapper Pro, which converted Sequoia images into a reflectance map. Given the setting of the albedo values for RCIs and using calibration parameters from the Parrot Sequoia sunshine sensor, radiometric calibration produced more reliable and accurate NDVI maps^1^. NDVI can be calculated using formula (2) as follows:

$$NDVI=\frac{\rho_{nir}-\rho_{red}}{\rho_{nir}+\rho_{red}}$$

To segment plants from soil, a concept proposed by Liebisch et al. (2015) was adopted as follows: using an NDVI threshold of 0.1 to discriminate non-plant and plant. A segmentation mask was produced by masking parts with an NDVI > 0.1 as plant while all other parts were soil. Four hundred and eighty-seven individual ROI_NDV I_ (ROI of NDVI map) were generated by overlaying ROI_plot_ and this mask using ArcGIS (version 9.3, Esri Inc., Redlands, CA, United States). A mean NDVI value within ROI_NDV I_ calculated suing ArcGIS zonal statistical analysis was employed as an NDVI representative value of each plot. CC was extracted as the ratio of the area between ROI_NDV I_ and ROI_plot_. According to this method, the time series NDVI and CC datasets were obtained for later dynamic analysis, respectively (Figure 1D).

### Data Validation and Statistics Analysis

**FIGURE 3:**
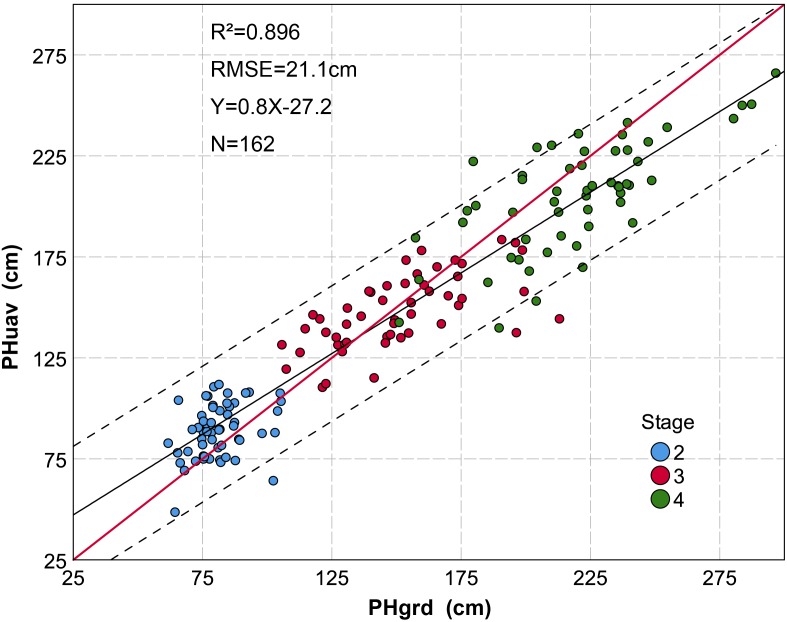
Grouped scatter plot of plant height derived from a UAV-HTPP (PHuav) *versus* ground truth ruler measurements (PHgrd). Data are based on a subset (removing the data that is affected by lodging) of sampling plots measured during three growth stages. The black solid line represents the regression line, the red solid line an implied 1:1 relationship, and the dotted line represents 95% confidence interval. *P*-value < 0.001 at the 0.01 level (two-tailed). A total of 162 samples were taken from three different growth stages. The discrete degree of plant height during different growth stages is different. Data are more discrete during later stages of growth.

Data validation and statistical analysis were completed using R software (version 3.2.4, R Core Team, 2016). In 72 sampling plots, PH was measured manually by a telescopic leveling rod during the S2, S3, and S4 stages. In order to reduce the influence of marginal effects and growth competition, the average of the three plants in the center of each sampling plot was counted as the representative value for the ground plant height (PHgrd). Before tasseling, the measurements were taken at 2/3 parts of the first expanded leaf. After tasseling, the measurements were taken on the top of the tassel. To validate the accuracy of the UAV plant height (PHuav) measurement, a linear regression model was applied with multiple dates (Figure 3). Parrot Sequoia multispectral camera relies on a sunshine sensor for automatic adjustment of readings to ambient light to minimize error during the imagery (Hassan et al., 2018), which enables multi-temporal NDVI to be comparable (see footnote 1).

Datasets from PH, CC, and NDVI were integrated by growth stage and genotypes. These datasets also contained some outliers that needed to be processed. For example, lodging makes PH decrease first and then increase, and the low emergence rate leads to an extremely low value of CC. Finally, 388 plots were further analyzed and discussed. Of these plots, 107 plots originated from GRP1, 28 plots originated from GRP2, 163 plots originated from GRP3 and 90 plots originated from the GRP4.

PH, CC, and NDVI represent potential indicators of growth traits. These traits in breeding programs are used to monitor genotypic differences and dynamic development. The purpose of this study is to obtain multi-temporal data from a UAV-HTPP to extract phenotypic traits among different genotypes and to conduct a comprehensive evaluation of the dynamic changes of traits and genotypic difference. The evaluation includes four aspects:

#### (1) Data distribution and phenotypic trait variation during different growth stages.

To explore trait data distribution, descriptive statistics and the Shapiro–Wilk test (SW test) were performed on three phenotypic trait data, according to the four growth stages. The SW test was applied to check whether continuous variables (traits) had a normal distribution. The test rejects the hypothesis of normality when the *p*-value is less than or equal to 0.05, and the data does not fit the normal distribution with 95% confidence. Skewness and kurtosis are used to describe the degree of distinctness between the data and data originating from a normal distribution. Mean, standard deviation (SD), coefficient variation (CV), and interquartile range (IQR) were used to describe the central tendency and dispersion of phenotypic traits. CV was used to analyze the degree of phenotypic trait variation during the different growth stages. When continuous variables obeyed a normal distribution, CV was calculated with mean and SD, otherwise it was calculated with median and SD.

#### (2) Correlation changes among multiple traits during different growth stages.

Using Pearson’s correlation coefficient to measure the linear relationship between two traits, and plotting the data to verify the linear relationship and to identify the potential outliers, we analyzed the dynamic changes of the relationship among multiple traits during the different growth stages.

#### (3) Extracting typical curves of dynamic change of a single trait using time series clustering analysis.

Cluster analysis was applied on time series trait datasets using the k-Shape clustering algorithm (Paparrizos and Gravano, 2016) to identify single trait mean dynamic change pattern, which is called a typical curve of dynamic changes. Distance measures provide quantification for the dissimilarity between two time series. The SBD was recently proposed as part of the k-Shape clustering algorithm (Sardá-Espinosa, 2017). The **dtwclust** package of R 3.2.4 provided this algorithm implementation and clustering quality evaluation. Davies–Bouldin Index (DBI) and Dunn Index (DI) were used as CVIs to determine the best clustering number, avoiding subjectivity in the selection of cluster number. The cluster number was set to 20 in advance, and then DBI and DI were iteratively calculated. The number of clusters most likely to minimize DBI and maximize DI simultaneously is considered as the optimal cluster number.

After clustering, a typical curve is generated by connecting the mean of the trait data during the four growth stages. A typical curve of a single trait represents a group of genotypes that has similar dynamic change.

## Results and Analysis

### Data Distribution and Phenotypic Trait Variation

**FIGURE 4:**
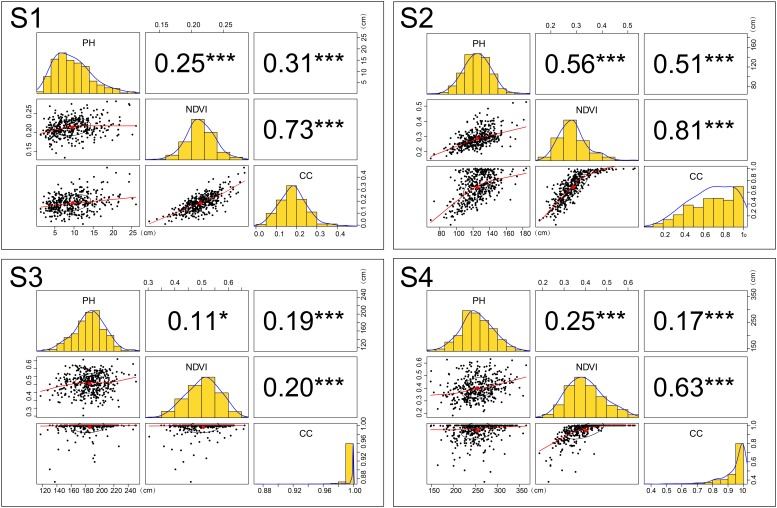
Traits (CC, NDVI, and PH) relationships and distributions during four different growth stages. Numerical values represent Pearson’s correlation coefficients between two traits. Asterisks indicate significant correlations using a two-tailed *t*-test (^∗∗∗^*P* < 0.001; ^∗^*P* < 0.05). Black ellipses represent correlation ellipses around the mean to reflect the correlation. The red line in the scatter plot represents a linear or non-linear regression line. Histograms and density plots reflect the distributions of different traits. CC, canopy cover; NDVI, normalized difference vegetation index; PH, plant height.

In Table 2, only five datasets passed the normality test (*p*-value > 0.05). In frequency histograms (Figure 4), when both kurtosis and skewness are close to 0, they can be regarded as approximately obeying normal distribution. Accordingly, we draw the following conclusions. CC does not obey normal distribution during all growth stages. During S3, kurtosis reached 41.87 and skewness -5.87, indicating that the distribution peaked more than the corresponding normal curve and the data are skewed to the left having a long tail that extends to the left with more extreme values. The degree of variation in CC was significant during S1 and S2, more than 30%. During S3 and S4, CV decreased rapidly, to less than 10%. This indicates that the indicative function of CC for crop growing is better during early stages than later. Excluding during S1, the PH data obey normal distribution. The CV of PH gradually decreased, which showed that the heterogeneity of PH among genotypes also gradually decreased. During S1 and S3, NDVI obeyed normal distribution. The CV of NDVI remained greater than 20% and the symmetry was worse during S2 and S4. Especially during the later stage, the increase in CV may show that genotypic difference occurs during the time to enter reproductive growth. In conclusion, the change in CV is a reflection to the variation in the traits with crop growth. The details of the trait data distribution are listed in Table 2.

**Table 2:** Descriptive statistics, normal distribution, and difference in three phenotypic traits during four growth stages.

Stage	Trait	Mean	SD	Minimum	Maximum	CV	Q1	Q2	Q3	Skewness	Kurtosis	*W*-test
S1	PH	9.62	4.77	0	26	53.0	6	9	12	0.81	0.42	0.000
	NDVI	0.21	0.02	0.13	0.28	9.5	0.20	0.21	0.23	0.09	0.65	0.064
	CC	0.18	0.07	0.09	0.48	38.9	0.13	0.18	0.22	0.59	1.21	0.000
S2	PH	124.99	16.70	69	184	13.4	114	125	136	0.09	0.64	0.126
	NDVI	0.29	0.06	0.15	0.53	21.4	0.25	0.28	0.31	0.89	1.43	0.000
	CC	0.68	0.22	0.05	1.00	31.4	0.52	0.70	0.87	-0.43	-0.68	0.000
S3	PH	185.98	21.51	117	251	11.6	173	187	200	-0.19	0.28	0.071
	NDVI	0.51	0.06	0.30	0.66	11.8	0.46	0.51	0.55	-0.22	-0.31	0.062
	CC	1.00	0.01	0.05	1.00	1.0	0.99	1.00	1.00	-5.87	41.87	0.000
S4	PH	253.45	36.80	148	365	14.5	231	251	279	0.10	0.14	0.450
	NDVI	0.40	0.09	0.18	0.63	23.0	0.34	0.39	0.45	0.38	-0.28	0.000
	CC	0.94	0.09	0.38	1.00	9.2	0.91	0.98	1.00	-2.51	8.06	0.000


*Significant at *P* < 0.05 using a two-tailed *t*-test, if *P* < 0.05, rejecting the null hypothesis, and there is significant difference between the data and that originating from a normal distribution. CC, canopy cover; NDVI, normalized difference vegetation index; PH, plant height; SD, standard deviation; CV, coefficient of variation; Q1, quantile 25%; Q2, quantile 50%; Q3, quantile 75%; W-test, Shapiro–Wilk test.*

### Correlativity Changes

Although the CC was derived from the NDVI calculation, the correlation between them is unstable. We observed a better correlation during the early stages (S1:0.73; S2:0.81). The Pearson’s correlation coefficient between PH and NDVI was all less than 0.6 during the different growth stages. The best correlation between the two is found during S2, when vegetative growth was vigorous. A similar situation existed for the correlation between PH and CC. Low correlation between PH and NDVI (or CC) indicated that when a maize yield multiple linear regression model is established, PH and NDVI (or CC) can work together as two independent predictor variables. Geipel et al. (2014) adopted this modeling approach. During S2, although Pearson’s correlation coefficient between CC and NDVI reached 0.81, the relationship between the two variables is obviously non-linear in the scatter plot; thus, the correlation coefficient might not be the best way to estimate the strength of the relationship. Pearson’s correlation coefficient is 0.56 during S2, but in the scatter plot with associated NDVI and PH, some scattered points deviate greatly from the fitting line (Figure 4).

### Typical Curves of Trait Dynamic Changes

**FIGURE 5:**
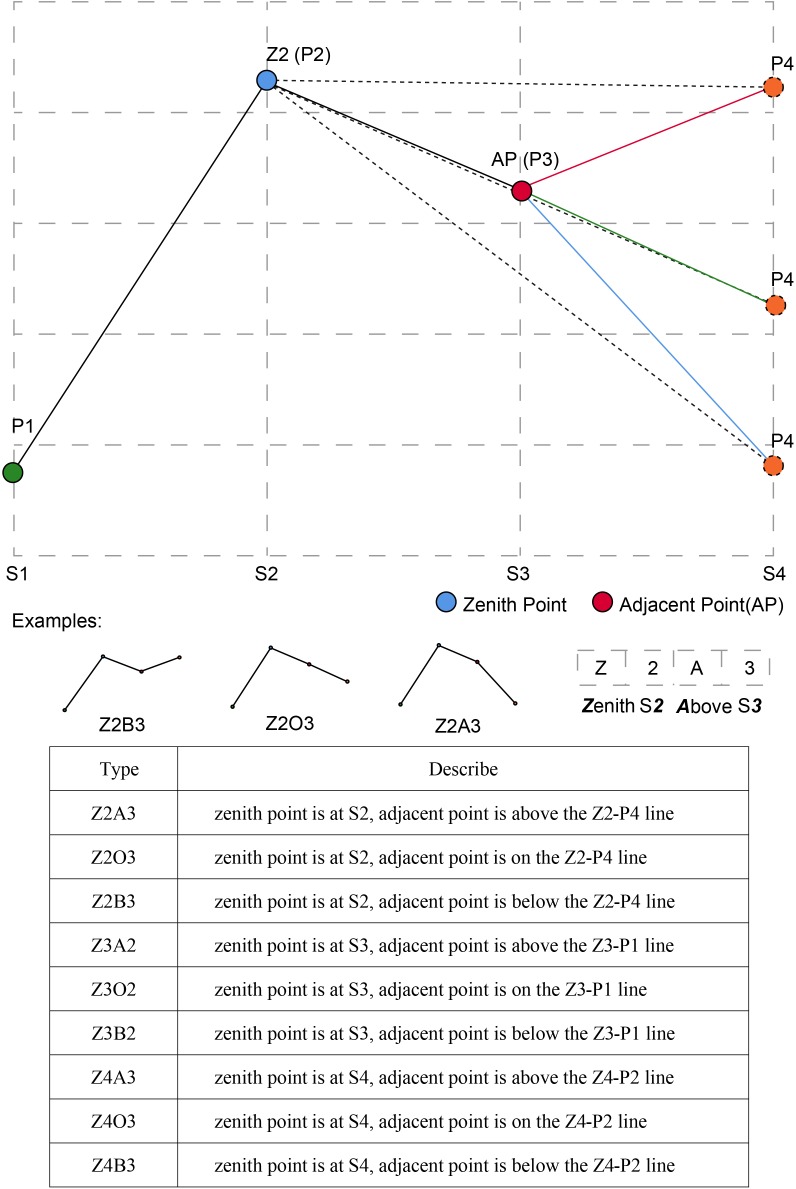
The naming convention of different typical curves and examples in this study.

Using time series clustering analysis, genotypes with a similar phenotypic trait change trend during different growth stages were clustered together, which was convenient for distinguishing the dynamic expression of the target trait in the time dimension. Typical curves showed the difference in shape and value of multi-temporal phenotypic trait clustering results. To facilitate the description and interpretation of the clusters, we created a naming convention to classify and name typical curves of these traits based on curve morphological feature (Figure 5), such as zenith, slope and the relationship between points and lines. In this study, there were nine types of typical curves, and each type also contained some subtle differences.

Plot value of trait (i.e., PH, CC, NDVI, AGRPH, or CRPH) on the vertical *Y*-axis against growth stage (i.e., S1, S2, S3, and S4) on the horizontal *X*-axis. Of the four points (P1–P4), two points played a decisive role in naming, namely the zenith point and its adjacent point. For example, the zenith point (i.e., maximum point) lies at S2 growth stage, so it is called Z2. Its adjacent point located at S3 growth stage is above the Z2-P4 line, so it is called A3. Connect the name of Z2 and A3 to form the name of the typical curve, that is, Z2A3. By analogy, Z2O3-type has a zenith point at S2 growth stage and an adjacent point on the Z2-P4 line.

**FIGURE 6:**
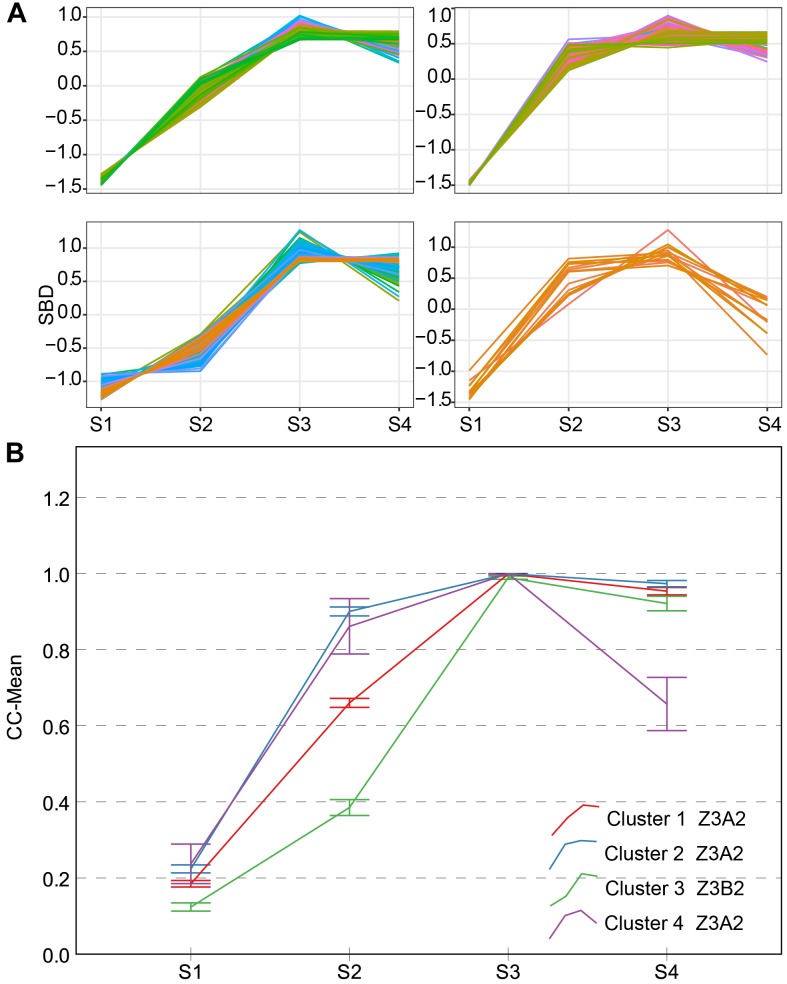
Clustering analysis for CC time series **(A)** and four clusters and typical curve of CC trait **(B)**. **(A)** The horizontal *X*-axis represents four growth stages, and the vertical *Y*-axis represents the shape-based distance. **(B)** The horizontal *X*-axis represents four growth stages, and the vertical *Y*-axis represents the mean value of CC at a specific growth stage. Zenith point lies at the S3 growth stage and its adjacent point lies at the S2 growth stage. Typical curves are based on mean values with a 95% confidence interval. CC, canopy cover; SBD, shape-based distance.

Two types of CC typical curves were named Z3A2 and Z3B2 (Figure 6). They all reach a maximum (approximate to 1) during S3, therefore the CV of CC during S3 is the lowest of all. The Z3A2-type contains three clusters, compared to cluster 3, cluster 1 and cluster 2 increased rapidly during S2 and did not decrease significantly during S4; whereas, cluster 4 decreased significantly during S4. They have higher level of leaf overlapping and more leaves during the early stage with greater vigor.

**FIGURE 7:**
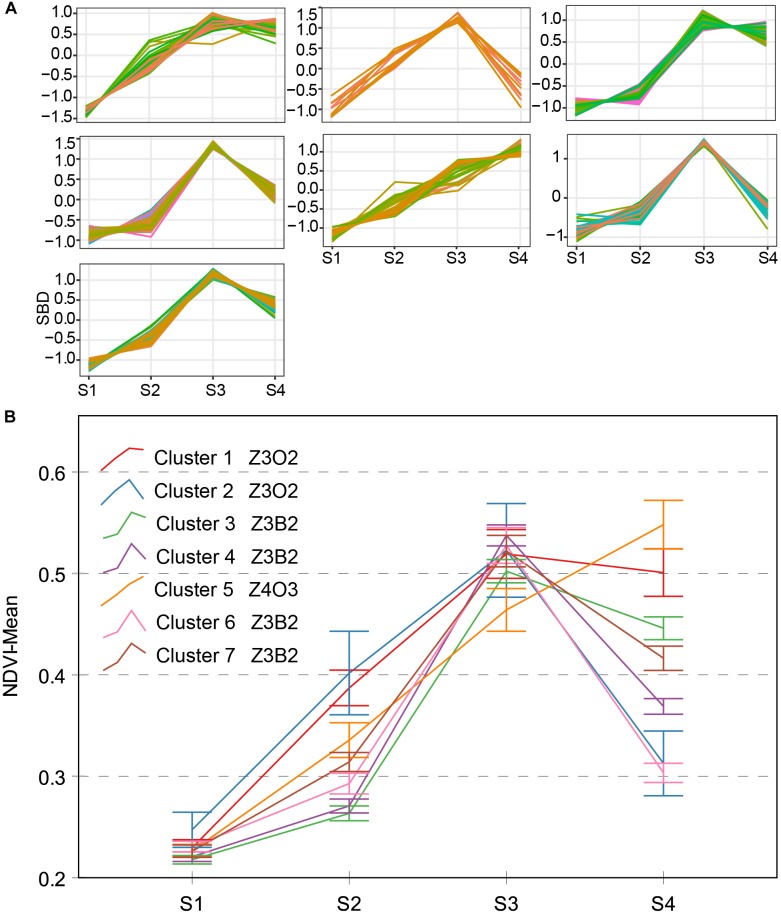
Clustering analysis for NDVI time series **(A)**, seven clusters and typical curve of NDVI trait **(B)**. **(A)** The horizontal *X*-axis represents four growth stages, and the vertical *Y*-axis represents the shape-based distance. **(B)** The horizontal *X*-axis represents four growth stages, and the vertical *Y*-axis represents the mean value of NDVI at a specific growth stage. Zenith point all lies at the S3 growth stage and its adjacent point lies at the S2 growth stage except cluster 5. Typical curves are based on mean values with a 95% confidence interval. NDVI, normalized difference vegetation index; SBD, shape-based distance.

Three types of NDVI typical curves were named Z3O2, Z3B2, and Z4O3 (Figure 7). The Z4A3-type contains only one cluster, which presented approximately straight-line growth. Different from the other type, the Z4O3-type strongly stays green even after flowering. A genotypic difference in phenology may lead to this result. Z3O2-type contains two clusters, and there is an obvious 0.19 of difference between them during S4. Z3B2-type contained four clusters and was comparable to other types with a trough point during S2. Excluding Z4A3-type, both the Z3O2-type and Z3B2-type decreased during S4. This is interesting and will be discussed further shortly. The Z3O2-type, Z3B2-type, and Z4O3-type showed differences in slope during S1–S3, which shows the inhomogeneous change of physiological and structural processes among different genotypes.

**FIGURE 8:**
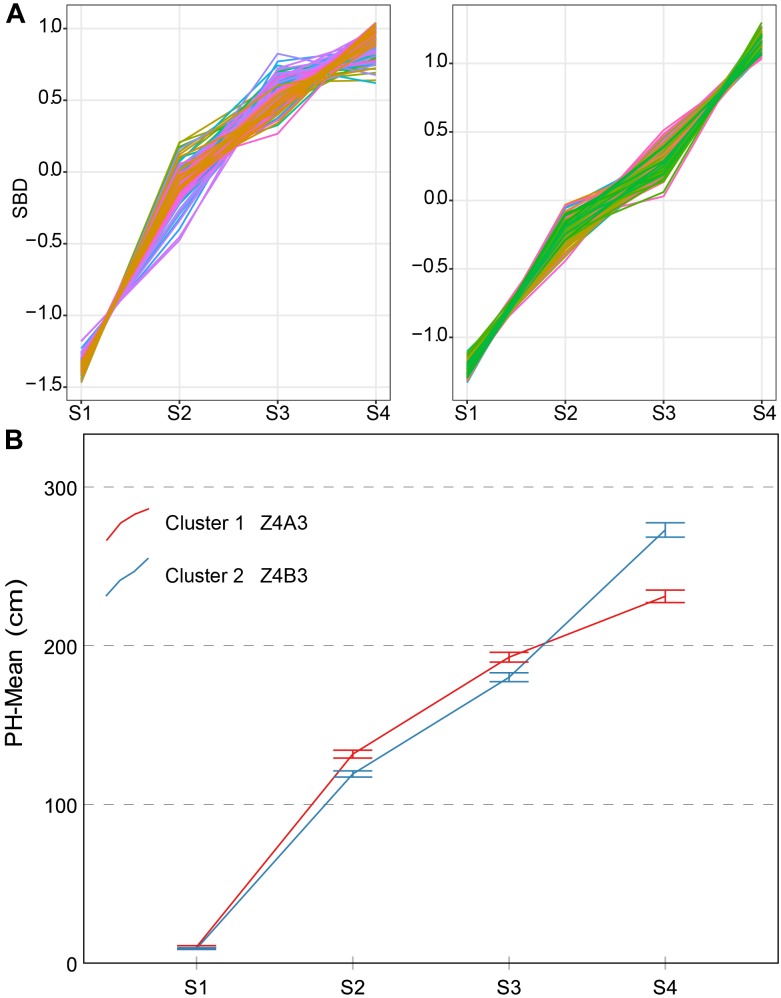
Clustering analysis for PH time series **(A)**, two clusters and typical curve for PH trait **(B)**. **(A)** The horizontal *X*-axis represents four growth stages, and the vertical *Y*-axis represents the shape-based distance. **(B)** The horizontal *X*-axis represents four growth stages, and the vertical *Y*-axis represents the mean value of PH at a specific growth stage. Zenith point lies at the S4 growth stage and its adjacent point lies at the S3 growth stage. Typical curves are based on mean values with a 95% confidence interval. PH, plant height; SBD, shape-based distance.

Two types of typical curves of PH were named Z4A3 and Z4B3 (Figure 8). From a confidence interval point of view, the time-series PH was well distinguished from the two clusters, but their shape characteristics were not. This may result in an inaccurate or inadequate sampling time point. We discovered that the average growth rate and the contribution rate of PH during different growth stages were better indicators. Their detailed descriptions are as follows:

Average Growth Rate:

$${AGRPH}_{G_{k}S_{i}}=\frac{{PH}_{G_{k}S_{i+1}}-{PH}_{G_{k}S_{i}}}{{Date}_{i+1}-{Date}_{i}}$$

Contribution Rate:

$${CRPH}_{G_{k}S_{i}}=\frac{{PH}_{G_{k}S_{i+1}}-{PH}_{G_{k}S_{i}}}{{PH}_{G_{k}S_{4}}}\times 100\%$$

Where subscript *G_k_* and *S_i_* represent k-th genotype and i-th growth stage, respectively. AGRPH is the ratio of PH increment to date increment between two adjacent growth stages, representing the absolute variation per day. CRPH is the percentage of PH increment to the final PH during different stages, reflecting the incremental distribution during different growth stages.

**FIGURE 9:**
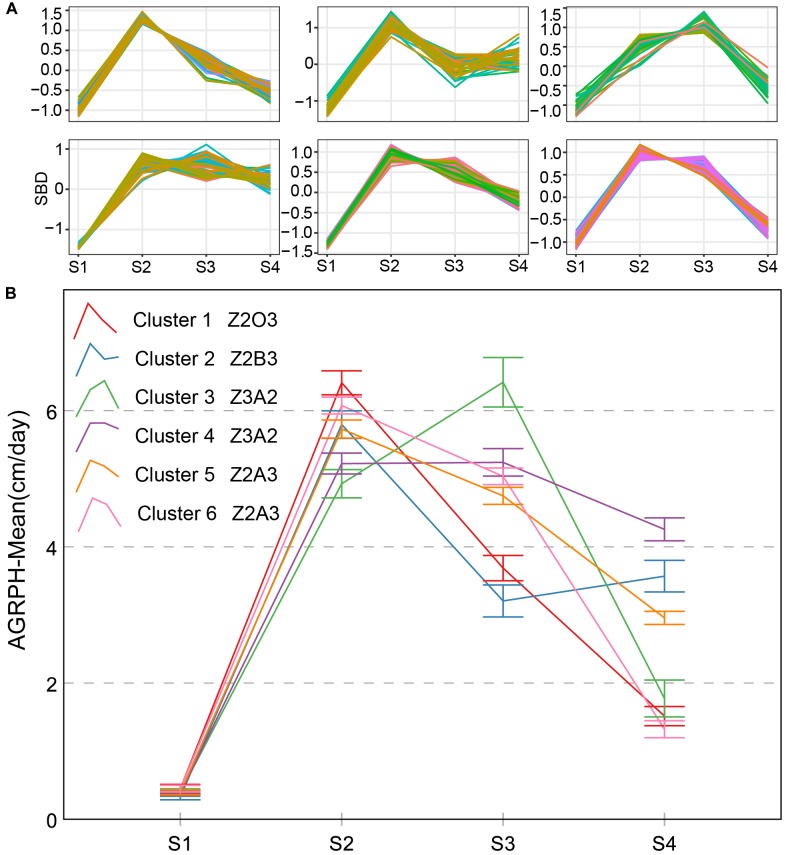
Clustering analysis for AGRPH time series **(A)** and six clusters and typical curve for AGRPH trait **(B)**. **(A)** The horizontal *X*-axis represents four growth stages, and the vertical *Y*-axis represents the shape-based distance. **(B)** The horizontal *X*-axis represents four growth stages, and the vertical *Y*-axis represents the mean value of AGRPH at a specific growth stage. In terms of cluster 1–2 and cluster 5–6, zenith point lies at the S2 growth stage and its adjacent point lies at the S3 growth stage. In terms of cluster 3–4, zenith point lies at the S3 growth stage and its adjacent point lies at the S2 growth stage. Typical curves are based on mean values with a 95% confidence interval. AGRPH, average growth rate of plant height; SBD, shape-based distance.

Four types of AGRPH typical curves were named Z2A3, Z2B3, Z2O3, and Z3A2 (Figure 9). Z3A2-type and Z2A3 contained two clusters, and the others contained one cluster, respectively. Z3A2-type had the highest average growth rate during S3 and one secondary peak during S2, which indicated that the cumulative increment of PH was the largest and vegetative growth was the most vigorous. The Z3A2-type showed a decreasing tendency and with a different value during S4 (mean is 8.8 and 11, respectively). Both the Z2A3-type and Z2B3-type had the highest average growth rate during S2, indicating rapid growth during the early stage. Z2B3-type first decreased during S3 and then rebounded during S4. It was possible that most genotypes contained in this cluster entered the reproductive growth stage later than the other genotypes.

**FIGURE 10:**
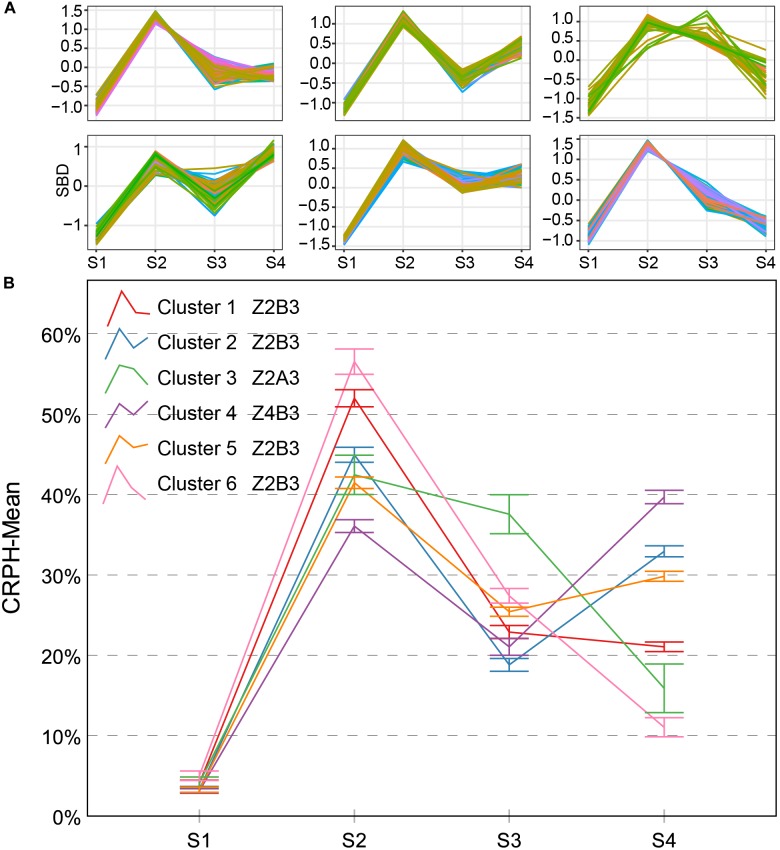
Clustering analysis for CRPH time series **(A)** and six clusters and typical curve for CRPH trait **(B)**. **(A)** The horizontal *X*-axis represents four growth stages, and the vertical *Y*-axis represents the shape-based distance. **(B)** The horizontal *X*-axis represents four growth stages, and the vertical *Y*-axis represents the mean value of AGRPH at a specific growth stage. Zenith point all lies at the S2 growth stage and its adjacent point lies at the S3 growth stage except cluster 4. Typical curves are based on mean values with a 95% confidence interval. CRPH, contribution rate of plant height; SBD, shape-based distance.

There are three types named Z2A3, Z2B3, and Z4B3 CRPH typical curves (Figure 10). There is an 89.2% overlap ratio between Z4B3-type and cluster 4 (Z3A2-type) of AGRPH, with the GRP3 group accounting for 78%. Combining these two clusters for analysis, during the stage from S3 to S4, AGRPH decreased from 14.0 to 11.0, and CRPH increased from 20.1 to 39.7. This shows that the maximum increment of PH and a longer time interval occurs during this period. All typical curves of CRPH reached a peak point during S2. This phenomenon is consistent with the common recognition that the most vigorous growth occurs during the growth stage 19 (BBCH-scale) (Lancashire et al., 1991).

A typical curve based on time-series trait clustering analysis can also be seen as a novel representative trait, which visually describes a group of genotypes with similar trends during different growth stages. As seen from the 95% confidence interval, the boundary of the typical curve is vague during S1 and S3, but clear during S4. Breeders also pay attention to during which stage trait difference is the highest or lowest. The F-statistics were calculated and sorted, and the preliminary conclusions are in Table 3.

**Table 3:** Trait difference between different growth stages.

Traits		S1	S2	S3	S4	Sorted
CC	F-statistic	8.538	14.973	4.427	2.025	S4-S3-S1-S2
	Sig.	^∗∗∗^	^∗∗∗^	0.001	0.074	
NDVI	F-statistic	5.536	69.402	7.678	124.611	S1-S3-S2-S4
	Sig.	^∗∗∗^	^∗∗∗^	^∗∗∗^	^∗∗∗^	
PH	F-statistic	5.280	62.556	36.188	184.266	S1-S3-S2-S4
	Sig.	^∗∗∗^	^∗∗∗^	^∗∗∗^	^∗∗∗^	
ARGPH	F-statistic	3.039	39.150	17.939	50.623	S1-S3-S2-S4
	Sig.	0.011	^∗∗∗^	^∗∗∗^	^∗∗∗^	
CRPH	F-statistic	11.062	172.670	114.190	519.663	S1-S3-S2-S4
	Sig.	^∗∗∗^	^∗∗∗^	^∗∗∗^	^∗∗∗^	


*Asterisks indicate significant correlations using a two-tailed *t*-test (^∗∗∗^*P* < 0.001). Not statistically significant indicates that clustering boundaries cross each other during this stage, and the trait difference is not obvious. Sorting is based on the effect of variables (stage) on clustering according to ascending order. CC, canopy cover; NDVI, normalized difference vegetation index; PH, plant height; AGRPH, average growth rate of plant height; CRPH, contribution rate of plant height.*

## Discussion

### Uncertainty Factor Analysis in Measurement

We have presented a method of extracting PH at the plot scale from a UAV-HTPP considering the spatial structure of the maize canopy. The core steps of this method are segmentation and spatial Kriging interpolation based on multiple neighboring maximum pixels from different plants in a plot. As a result, the PHuav is the interpolation of the top of multiple plants in a plot, covering the spatial distribution information of multiple plants. In this study, PHuav strongly correlates with the ground measurement (PHgrd) (*R*^2^ = 0.896) in the sampling plot during multiple growth stages. During the later growth stage, PHgrd is greater than PHuav more often. This may be because PHgrd was measured at the top of tassels after tasseling, while the size of the tassel varied with the plant population and variety (Duncan et al., 1967). If the tassel’s point cloud by remote sensing was too limited to completely reconstruct a tassel model, this would lead to smaller PH value. Maresma et al. (2016) and Shi et al. (2016) also reported that the length of the tassel may pose influence on the PH measurement. The underestimation of PH has also been reported for other crops, such as barley (Araus and Cairns, 2014; Bendig et al., 2014), wheat (Holman et al., 2016), sorghum (Watanabe et al., 2017), and vines (Matese et al., 2017), in accordance with our study. As previously mentioned, PH extracted from UAV images is influenced by the size of the tassel, and this leaves room for further improvements. One option would be, for example, to reduce flight altitude to ensure that tassels have enough point clouds.

Surprisingly, The NDVI trait of most genotypes decreased during S4 after increasing to a peak during S3. There are three reasons for the NDVI decrease as follows: fertilizer, canopy structure and water-stress. First, because the single-factor experiment limited fertilization throughout the growth stage, the lack of fertilizer caused the leaves of most genotypes to begin to senesce after entering reproductive growth. Second, in addition to leaves, the maize canopy also included tassel branches and flowerlets during the later growth stages, which may have led to NDVI change. Finally, from July 11th to August 7th, the field meteorological station recorded five intense rainfall in the experimental area. Therefore, water-stress may have led to premature senescence causing the NDVI to decrease.

In addition to selecting the most appropriate traits, it is also essential to determine the critical time for their evaluation (Araus and Cairns, 2014). High-throughput phenotyping timepoints for field traits of interest are critical for manageable data collection and analysis (Shakoor et al., 2017). The sampling timepoints may have an impact on the extraction of time-series traits, because these traits are in continuous change among 487 different genotypes throughout the growing season. ARGPH and CRPH are relatively more prone to time-scale problems.

Phenotypic traits measured by remote sensing are all on the canopy scale and are affected by observation angle, illumination conditions, canopy structure, and leaf morphological characteristics (Walter et al., 2015). Aasen and Bolten (2018) have conducted in-depth research on these issues. In theory, segmentation of plant from soil can provide more accurate results for phenotypic trait extraction. In this study, we adopted a simple threshold method to attain this end. Image-based automatic phenotyping acquisition technologies have made significant advances in recent years, Perez-Sanz et al. (2017) has given an overview of the image segmentation method.

### Genotypic Differences

**FIGURE 11:**
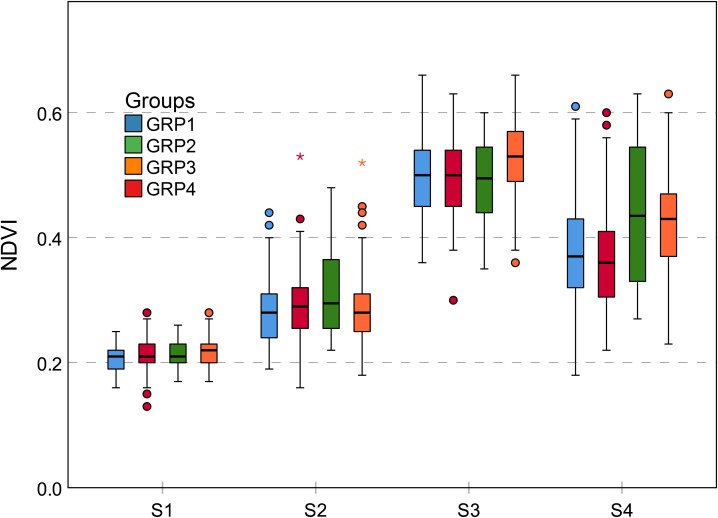
Grouped boxplot for genetic background of NDVI trait. Outliers are shown as dots and asterisks. The difference of NDVI between four groups is most obvious at the S4 growth stage. All groups achieved the largest NDVI at S3 growth stages. GRP2 and GRP3 have less NDVI decline at the S4 growth stage. NDVI, normalized difference vegetation index.

Genotype-by-environment interaction results in different phenotypic traits which are observable at the structural and physiological levels (Dhondt et al., 2013; Walter et al., 2015). NDVI is closely related to leaf color. Maize leaf color was controlled by genes. Leaf color variation is a common mutation trait, which is usually expressed during the seedling stage, but a few mutants do not change leaf color until the late growth stage, responding to environmental conditions (Zhong et al., 2015). For NDVI, leaf color differences and mutations may lead to a large number of typical curves and a rich phenotypic trait variation (Figure 7 and Table 2). Genetic background also affects trait expression. The GRP2 has the characteristic of lodging-resistance. When planted in temperate regions, GRP3 needs a prolonged growth period, reflecting the phenological differences. Thus, GRP2 and GRP3 can limit the reduction of leaf stay-green to a certain extent after flowering and under stress conditions (strong winds and heavy rain), which results in a relatively higher NDVI value during S4 (Figure 11). Cluster 5 is composed of 32 genotypes and has the highest NDVI mean value during S4 (Figure 7); however, it only contained 13 genotypes originating from the GRP3, accounting for less than 41%. This figure shows that the NDVI trait is more likely to be affected by the external environment.

**FIGURE 12:**
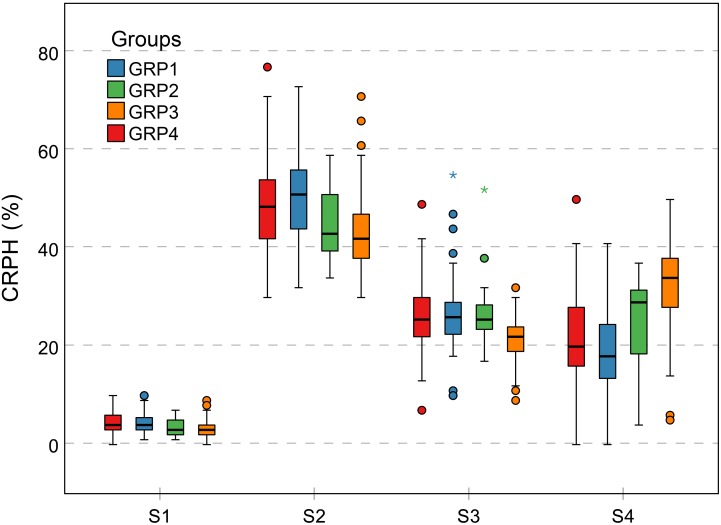
Grouped boxplot for genetic background of CRPH trait. Outliers are shown as dots and asterisks. The vertical *Y*-axis represents the CRPH grouped according to genetic background, and the horizontal *X*-axis represents four growth stages. The difference in CRPH between the four groups is relatively large at the S2 and S4 growth stages. CRPH, contribution rate of plant height.

**FIGURE 13:**
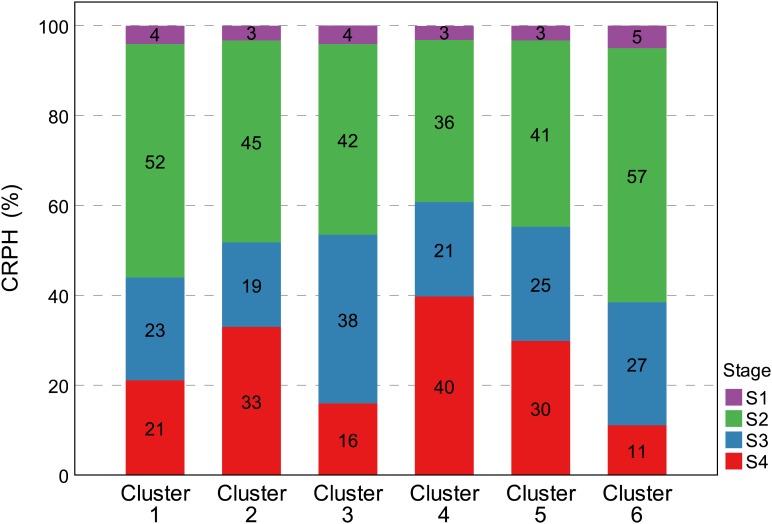
Percentage distribution of CRPH trait in the six clusters during four growth stages. The vertical *Y*-axis represents the percentages distribution of CRPH at four growth stages, and the horizontal *X*-axis represents six clustering results of CRPH typical curve. The cumulative value of the *Y*-axis is 100%. All groups achieved the largest CRPH at the S2 growth stages. Cluster 2 and cluster 4 still retained higher plant height increment at the S4 growth stage. CRPH, contribution rate of plant height.

Contribution rate of plant height is a relative increment based on the final PH, focusing on individual difference in genotypes. This trait can effectively indicate the difference between genetic backgrounds during different growth stages. As previously mentioned, because the GRP3 growth period was prolonged, the CRPH was the lowest in general during the first three growth stages, while the highest was during S4 compared to the other groups (Figure 12). This phenomenon showed that the development of the GRP3 lagged, while the other groups had entered reproductive growth, it was still in vegetative growth. Cluster 4 and cluster 2 (Figures 10, 13) can represent this phenomenon. Cluster 4 contains 68 genotypes, of which 56 originated from the GRP3, accounting for 82.4% in the cluster. Cluster 2 contains 63 genotypes, of which 44 originated from the GRP3 population, accounting for 69.8% in the cluster. Compared to the NDVI, the difference between the genetic backgrounds of PH is more significant.

## Conclusion

We developed a semi-automated pipeline for analyzing and evaluating multiple phenotypic traits (CC, NDVI, PH, AGRPH, and CRPH) derived from a UAV-HTPP, and introduced a time series data clustering analysis method into breeding programs as a tool to obtain a novel representative trait: typical curve. Furthermore, we identified and evaluated in detail genotypic differences and dynamic changes during different maize growth stages. We found that typical curves can detect a difference in the genetic background of traits. The recognition rate of the NDVI typical curve is 59%, far less than the CRPH typical curve of 82.4%. Our study provides evidence that the PH trait is among the most heritable and the NDVI trait is among the most easily affected by the external environment in maize. We have verified that a UAV-HTPP can offer an efficient, non-invasive, flexible and low-cost solution for large-scale breeding programs compared to ground investigation, especially where measurements are time-sensitive.

## Author Contributions

BX performed the remote sensing experiments. LH and HY organized and collected the on-ground and aerial data. ZL, XY, and GY obtained funding for the study. LH performed the statistical analyses, interpreted results, and drafted the manuscript. GY supervised the study. All authors read and approved the final manuscript.

## Conflict of Interest Statement

The authors declare that the research was conducted in the absence of any commercial or financial relationships that could be construed as a potential conflict of interest. The handling Editor is currently co-organizing a Research Topic with one of the authors GY and confirms the absence of any other collaboration.

## Footnotes

1 https://www.micasense.com
